# XenoMix: estimating mouse content from DNA methylation data of Patient-Derived Xenograft models

**DOI:** 10.1093/bioinformatics/btag791

**Published:** 2026-10-21

**Authors:** Nima Esmaeelpour, Oliver Mücke, Gizem Altun, Joseph Cornick, Maria Capone, Rocio Sotillo, Maria Llamazares-Prada, Christoph Plass, Michael Scherer

**Affiliations:** Division of Cancer Epigenomics, German Cancer Research Center (DKFZ), Heidelberg, Germany; Division of Cancer Epigenomics, German Cancer Research Center (DKFZ), Heidelberg, Germany; Division of Cancer Epigenomics, German Cancer Research Center (DKFZ), Heidelberg, Germany; Faculty of Biosciences, Heidelberg University, Heidelberg, Germany; Division of Cancer Epigenomics, German Cancer Research Center (DKFZ), Heidelberg, Germany; Faculty of Biosciences, Heidelberg University, Heidelberg, Germany; Division of Molecular Thoracic Oncology, German Cancer Research Center (DKFZ), Heidelberg, Germany; Division of Molecular Thoracic Oncology, German Cancer Research Center (DKFZ), Heidelberg, Germany; Division of Cancer Epigenomics, German Cancer Research Center (DKFZ), Heidelberg, Germany; Division of Cancer Epigenomics, German Cancer Research Center (DKFZ), Heidelberg, Germany; Division of Cancer Epigenomics, German Cancer Research Center (DKFZ), Heidelberg, Germany

## Abstract

**Summary:**

DNA methylation microarrays enable cost-effective, high-throughput profiling of human and mouse samples and are widely used to study epigenetic alterations in cancer. Patient-Derived Xenograft (PDX) models complement this approach by preserving primary tumor characteristics while enabling *in vivo* studies of drug response and therapy resistance. However, it remains difficult to quantify mouse DNA contamination in PDX DNA methylation data generated using microarrays. We present XenoMix, a light-weight computational tool for quantifying mouse DNA contamination directly from DNA methylation data in PDX models. XenoMix leverages the high sequence homology between human and mouse genomes to detect and quantify unexpected signal contributions originating from mouse DNA. Using ground truth mixtures, XenoMix achieved Pearson’s correlation coefficients higher than 0.99. When applied to PDX data, we identified varying levels of mouse contamination originating from biological and technological variability. XenoMix establishes a critical quality control step in DNA methylation analysis of PDX models, and we show that the estimated contamination can be incorporated as a confounding variable in downstream analyses.

**Availability and implementation:**

XenoMix is available as a standalone R package from https://git.dkfz.de/b370/scepigen/XenoMix and as a module in the RnBeads software package.

## 1 Introduction

Many cancer types are characterized by profound inter and intra-patient heterogeneity. In vitro cancer models insufficiently reflect the complexity of cancer and fail to capture the effects of the tumor microenvironment. Patient-Derived Xenografts (PDX) preserve primary tumor characteristics, including genetic, histological and molecular heterogeneity, while providing a functional *in vivo* environment to study systemic interactions (Blanchard *et al*. 2025). These models are established by transferring tumor cells from a patient into immunodeficient or humanized recipients, mostly mice (Fig. 1a). Tumor cells can engraft in the host environment and recapitulate human tumor biology, including tumor progression and evolution (Abdolahi *et al*. 2022, Liu *et al*. 2023). PDX models enable the exploration of drug-resistance mechanisms and the molecular profiling of tumors, for example by profiling DNA methylation (Gómez-Miragaya *et al.* 2019, Trnkova *et al*. 2026). The most widely applied method for DNA methylation profiling is the Illumina Infinium microarray series, where in the newest version (EPICv2), close to one million CpGs can be profiled at high throughput and accuracy. However, tumor purity and mouse contamination are crucial factors to be considered when analyzing molecular profiles such as DNA methylation in PDX models (Jo *et al*. 2019, Sasiain *et al*. 2024). Various computational and experimental tools have been developed to either remove or account for murine contamination, focusing primarily on transcriptomic and genomic sequencing data (Conway *et al*. 2012, Zentgraf and Rahmann 2021, Bhandari *et al*. 2025), but not on DNA methylation data obtained by microarrays. Here, we present XenoMix, the first tool to estimate murine contamination directly from DNA methylation microarray data. We validated XenoMix using artificial mixtures of human and mouse DNA. When applied to published PDX data, we found varying levels of mouse contamination across different cancer models that can be corrected for in downstream analysis.

**Figure 1:**
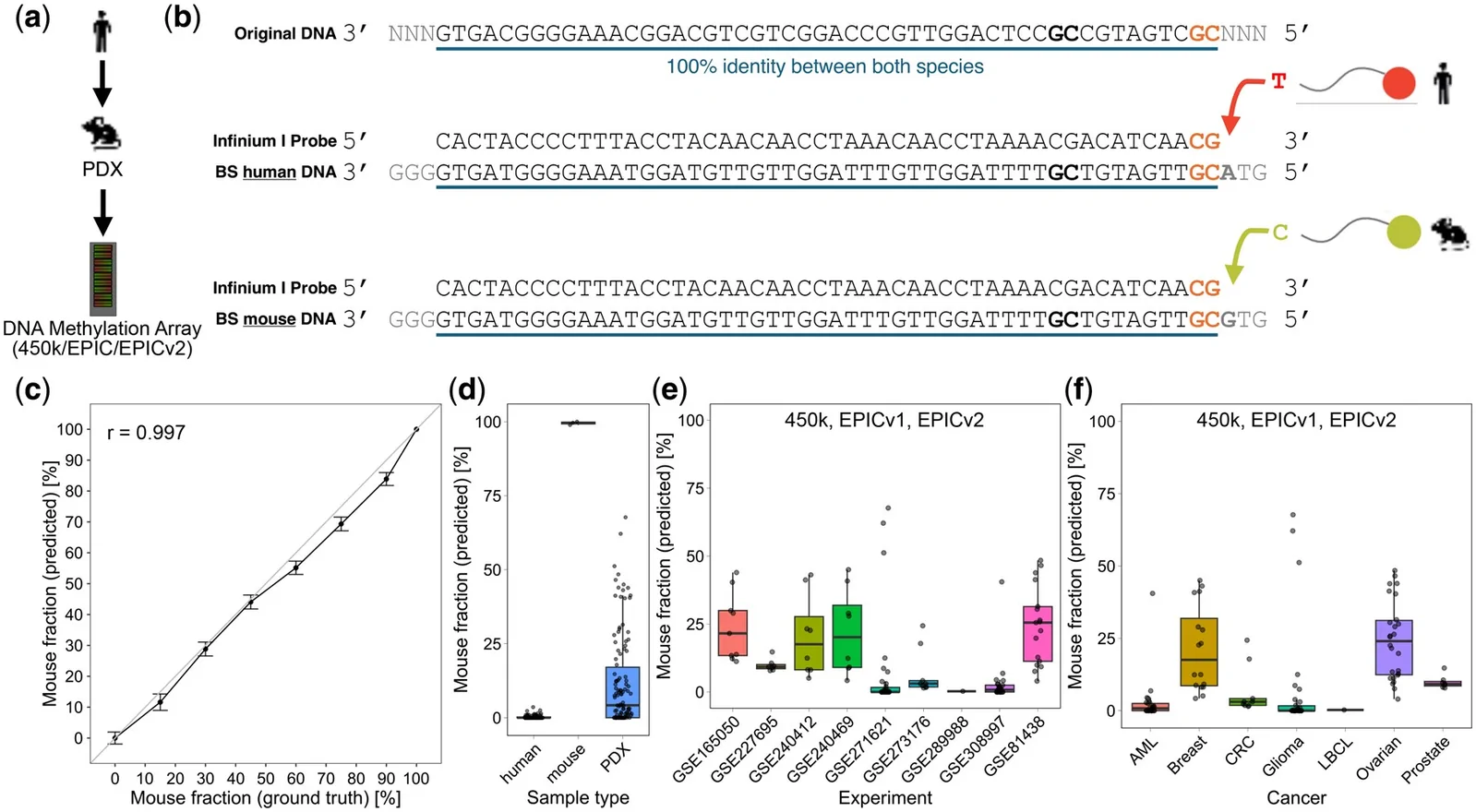
Development and validation of XenoMix. (a) Schematic overview of the Patient-Derived Xenograft (PDX) workflow. (b) Rationale of inter-species informative probe selection. XenoMix exploits probes on DNA methylation microarrays, where the extension bases, and their resulting color channels, differ between the two species. (c) Comparison between predicted and ground-truth mouse DNA fractions using human-mouse mixtures for eight different mixtures. The symbol *r* represents the Pearson correlation coefficient. (d) Predicted mouse fractions across 249 external DNA methylation datasets from GEO, stratified by sample type (Human, PDX, and Mouse). (e, f) Estimated contamination across individual PDX experiments stratified by dataset (e) and cancer type (f). This figure has been designed using resources from Flaticon.com by users G-CAT (https://www.flaticon.com/free-icon/rat_12634989) and DinosoftLabs (https://www.flaticon.com/free-icon/man_3439472).

## 2 Implementation

### 2.1 Inter-species contamination detection using Infinium microarrays

XenoMix leverages the high sequence homology between mouse and human genomes and screens for probes on DNA methylation microarrays with perfectly matching sequences between the two species, inspired by Zhou *et al*. (2022). To identify such probes in the Illumina Infinium BeadArrays, we extracted the 50 bp probe sequence and aligned them to the mm10 mouse reference genome. Both strand orientations were evaluated and only mismatch-free, full-length, and uniquely mapping (mouse genome mm10) matches were considered. We excluded probes overlapping with any known single nucleotide polymorphisms in either the human (hg38) or mouse (mm10) genomes. We exclusively used Type I probes, which use two designs (a methylated [M] and unmethylated [U]) for each target CpG sequence and measure DNA methylation through the signal emitted from the subsequent base. In the original design, there are two template sequences per genomic locus: one complementary to an unmethylated CpG at the end of a probe (i.e. a CA) and one complementary to a methylated CpG (i.e. a CG). The color of the subsequent nucleotide is not used for inferring methylation states, but can be leveraged for identifying the subsequent base (A/T or C/G). For each Type I probe, the expected human color channel was extracted from Illumina’s manifest file. The corresponding expected extension color in mouse was then inferred by determining the base immediately following the targeted CpG, accounting for bisulfite-conversion (cytosines converted to thymines). Finally, probes were classified as suitable for inter-species discrimination if the inferred mouse color channel disagreed with the expected human color (Fig. 1b). We identified 15 such probes present on the Infinium microarrays (450k/EPIC/EPICv2) that fulfilled our criteria and can thus be used for estimating mouse contamination.

Mouse DNA contamination was quantified from inter-species probe intensities by exploiting discordant red/green signals. Type I probe methylated and unmethylated intensities in the red (R) and green (G) channels were extracted per sample from raw intensity data (IDAT) files using the SeSAMe R package (Zhou *et al*. 2018), yielding the signals $M_{R},\;U_{R},\;M_{G},\;U_{G}$ , respectively. For each probe [*i*] the total signal $S_{i}$ was defined as


$$S_{i}=M_{R,i}+U_{R,i}+M_{G,i}+U_{G,i}$$

Only probes with $S_{i}>1000$ were retained to reduce noise. The expected human color signal was used to compute a per-probe wrong-color fraction $w_{i}$.


$$w_{i}=\frac{M_{R,i}+U_{R,i}}{S_{i}}$$

when the expected channel was green, and


$$w_{i}=\frac{M_{G,i}+U_{G,i}}{S_{i}}$$

when the expected channel was red.

A sample-specific background level *b* was estimated as the median $w_{i}$ across all Type I probes, representing baseline color leakage and other non-specific effects. The raw mouse contamination signal *m* was then computed as the median $w_{i}$ of the retained inter-species informative probes. The final mouse fraction estimate $\hat{f}\epsilon [0,\;1]$ applied conditional background correction:


f^={max⁡(0, m-b), if m<0.4m, if m≥0.4

This rule was chosen empirically from the ground-truth mixture series, where subtraction of *b* improved calibration at low mouse fractions. Uniform subtraction across the full range led to overcorrection at higher mouse fractions (Fig. S1, available as supplementary data at *Bioinformatics* online). Details of the calibration procedure and the observed *b* values for all mixtures are provided in Table S1, available as supplementary data at *Bioinformatics* online.

### 2.2 Generation of human-mouse mixtures

To generate a ground-truth dataset with known human and mouse content, we mixed eight different fractions of human and mouse DNA (Table S2, available as supplementary data at *Bioinformatics* online). Human DNA was obtained from the A549 cell line and mouse DNA from a cell line derived from a lentiviral Cre-induced KPC (KrasG12D/+/p53 fl/fl) (DuPage *et al*. 2009) and (LSL- Rosa26LSL-Cas9-EGFP) (Platt *et al*. 2014) tumor. The mixture was hybridized on the Illumina Infinium EPICv2 BeadArray at the Microarray Core Facility of the German Cancer Research Center.

### 2.3 Availability

XenoMix is available as a standalone R package distributed under the GNU GPL v3 license (https://git.dkfz.de/b370/scepigen/XenoMix), from Figshare (https://doi.org/10.6084/m9.figshare.32715468), and as a module within the RnBeads R package (Müller *et al*. 2019). The data from the artificial human/mouse mixtures are available from ArrayExpress under accession number E-MTAB-16743.

## 3 Results

XenoMix quantifies murine contamination in PDX models by leveraging “inter-species informative probes” on DNA methylation microarrays that target genomic regions with perfect sequence homology between human and mouse. When the base immediately following the targeted CpG site is different between the two species, the resulting single-base extension emits a discordant color signal. A systematic screen of the Infinium 450k/EPICv1/v2 array identified 15 specific inter-species probes meeting our filtering criteria (Methods, Fig. 1b, Table S3, available as supplementary data at *Bioinformatics* online). Performing the same screen on the Infinium mouse array, we identified 11 such probes in line with the publication introducing the mouse array and the concept of cross-species probes (Zhou *et al*. 2022) (Table S4, available as supplementary data at *Bioinformatics* online). XenoMix uses the color intensities of these probes to estimate mouse contamination from PDX samples. We evaluated the performance of XenoMix across eight artificial human-mouse DNA mixtures (ranging from 0% to 100% mouse DNA). We observed a strong correlation between ground-truth mouse fraction and XenoMix’ prediction (0.997, Fig. 1c). We further evaluated XenoMix’ performance using 249 publicly available samples from the GEO database, spanning various tissues and cancer types (Table S5, available as supplementary data at *Bioinformatics* online). XenoMix correctly identified human-only samples as having near-zero contamination (Fig. S2, available as supplementary data at *Bioinformatics* online), while mouse-only samples were estimated at around 100% (Fig. 1d). In published datasets of PDX samples, we observed a wide range of murine contamination, with some samples exceeding 50% of contaminating mouse DNA. Our analysis revealed substantial inter-study and intra-study variability in host DNA content (Fig. 1e), likely reflecting differences in tumor entity (Fig. 1f), sample preparation, or passage number. We confirmed that XenoMix is compatible with data generated using the 450k, EPICv1 and EPICv2 microarrays (Fig. S3, available as supplementary data at *Bioinformatics* online). Finally, we used XenoMix’ estimated mouse fractions to perform in silico purification of DNA methylation data of breast and colorectal carcinoma PDX models. We found that after purification of the putative mouse content using PureBeta (Sasiain *et al*. 2024), the estimated fraction of epithelial cells as the cell-of-origin of carcinomas is increased (Fig. S4, available as supplementary data at *Bioinformatics* online). In contrast, putative mouse cell type proportions (fibroblasts, immune cells) were decreased. This indicates that in silico purification of PDX DNA methylation data using XenoMix’ estimated mouse fractions is feasible.

## 4 Discussion

PDX models retain the genomic and epigenetic features of the original tumor and allow for the investigation of drug efficacy and resistance mechanisms (Yoshida 2020). However, the fidelity of any downstream molecular analysis, particularly in epigenetic profiling, depends on tumor purity. A major technical hurdle in PDX research is host contamination, where murine stromal cells from the tumor microenvironment are co-harvested with the human tumor. While DNA methylation arrays are widely used for PDX profiling due to their cost-efficiency and standardization, they lack a built-in mechanism for species discrimination. Traditional methods to quantify mouse content, such as PCR-based approaches (Jin *et al*. 2023), require additional experimental work and sample availability. In contrast, XenoMix provides a retrospective in silico solution, quantifying murine content directly from existing DNA methylation array data. XenoMix allows for the re-evaluation of publicly available datasets where physical material is no longer accessible. While the concept of leveraging Type I probe color channels for species detection was explored in a human-on-mouse context (Zhou *et al*. 2022), XenoMix is the first tool specifically dedicated to quantify host DNA contamination within human methylation datasets. Compared to existing tools for estimating host DNA/RNA in sequencing data (Conway *et al*. 2012, Zentgraf and Rahmann 2021), XenoMix leverages data generated by widely used and cost-effective microarrays. In PDX samples, we found a broad range of host DNA estimates, with some outliers exceeding 50%. This high variability in murine content is likely driven by several biological and experimental factors, including sorting prior to hybridization or different tumor types. The individual performance of the 15 identified inter-species informative probes varied substantially. We solved this issue by computing their median and applied background correction. Despite these adjustments, a small number of human samples yielded marginal, non-zero contamination estimates. It remains unclear whether these outliers result from technical or biological variation. Host contamination below 10% likely does not compromise DNA methylation data quality (Samuels *et al*. 2010), and in these cases XenoMix can serve as a quality control step. For samples with substantial contamination, XenoMix provides the necessary data to add the predicted mouse fraction as a covariate in a differential analysis, or to use it for in silico purification (Sasiain *et al*. 2024).

## Supplementary Material

btag791_Supplementary_Data
