# Supplementary material for: XenoMix: estimating mouse content from DNA methylation data of Patient-Derived Xenograft models
Source: Bioinformatics. 2026 Oct 21;42(11):btag791. doi: 10.1093/bioinformatics/btag791 (PMC13725749; doi:10.1093/bioinformatics/btag791)
Supplement: Supplementary file 1 [file btag791_supplementary_data.zip › SupplementaryMaterial.docx]

**Supplementary Material to:**

**XenoMix: Estimating mouse content from DNA methylation data of Patient-Derived Xenograft models**

Supplementary Figures

**Figure S1:** Calibration of the background correction method using the ground-truth human/mouse mixture as a reference. (a) Estimated mouse fractions compared to the ground-truth without using background. (b) Estimated mouse fractions compared to the ground-truth using background corrections across the full range of estimated fractions. In the final version of XenoMix, background correction is only performed until an estimated value of 0.4 mouse contamination (see main Figure, panel C).

**Figure S2:** Estimated mouse contamination values for GEO datasets from DNA methylation data generated from human samples. (b) shows the same data, zoomed in on the area between 0 and 5%.

**Figure S3:** Comparison of estimated mouse contamination between 450k, EPICv1 and EPICv2 datasets. The collected GEO datasets (Table S3) were stratified into 450k, EPICv1 and EPICv2, and then according to the sample type (human and PDX). XenoMix’ estimated mouse content is visualized as boxplots.

**Figure S4:** Cell type proportions of epithelial cells, fibroblasts, B cells, NK cells, and monocytes as estimated using EpiDISH before and after correction of the beta value matrix with PureBeta using XenoMix’ estimated mouse fractions. The plot shows PDX DNA methylation data (EPICv2) from breast adenocarcinoma (GSE240412) and colorectal carcinoma (GSE273176) from samples with non-zero mouse cell types (fibroblast or immune cells) estimated before correction.

Supplementary Methods

**In silico purification of PDX data**

To show how the estimated mouse content by XenoMix can be used for in silico purification of DNA methylation data from PDX models, we used the PureBeta R package using the estimated mouse content as the variable to be corrected for. We then computed cell type proportions using EpiDISH before and after the correction with PureBeta using the built-in references for epithelial, fibroblasts and three immune cells (B cells, monocytes, and NK cells). This was performed for EPICv2 data from breast (GSE240412) and colorectal carcinoma (GSE273176), since these were
